# Identification of GPCR-Interacting Cytosolic Proteins Using HDL Particles and Mass Spectrometry-Based Proteomic Approach

**DOI:** 10.1371/journal.pone.0054942

**Published:** 2013-01-25

**Authors:** Ka Young Chung, Peter W. Day, Gisselle Vélez-Ruiz, Roger K. Sunahara, Brian K. Kobilka

**Affiliations:** 1 Department of Molecular and Cellular Physiology, Stanford University, Medical School, Beckman Center, Stanford, California, United States of America; 2 School of Pharmacy, Sungkyunkwan University, Jangan-gu, Suwon, South Korea; 3 Biological Technologies, Genentech, Roche Group, South San Francisco, California, United States of America; 4 Department of Pharmacology, University of Michigan Medical School, Ann Arbor, Michigan, United States of America; Loyola University Chicago, Stritch School of Medicine, United States of America

## Abstract

G protein-coupled receptors (GPCRs) have critical roles in various physiological and pathophysiological processes, and more than 40% of marketed drugs target GPCRs. Although the canonical downstream target of an agonist-activated GPCR is a G protein heterotrimer; there is a growing body of evidence suggesting that other signaling molecules interact, directly or indirectly, with GPCRs. However, due to the low abundance in the intact cell system and poor solubility of GPCRs, identification of these GPCR-interacting molecules remains challenging. Here, we establish a strategy to overcome these difficulties by using high-density lipoprotein (HDL) particles. We used the β_2_-adrenergic receptor (β_2_AR), a GPCR involved in regulating cardiovascular physiology, as a model system. We reconstituted purified β_2_AR in HDL particles, to mimic the plasma membrane environment, and used the reconstituted receptor as bait to pull-down binding partners from rat heart cytosol. A total of 293 proteins were identified in the full agonist-activated β_2_AR pull-down, 242 proteins in the inverse agonist-activated β_2_AR pull-down, and 210 proteins were commonly identified in both pull-downs. A small subset of the β_2_AR-interacting proteins isolated was confirmed by Western blot; three known β_2_AR-interacting proteins (Gsα, NHERF-2, and Grb2) and 3 newly identified known β_2_AR-interacting proteins (AMPKα, acetyl-CoA carboxylase, and UBC-13). Profiling of the identified proteins showed a clear bias toward intracellular signal transduction pathways, which is consistent with the role of β_2_AR as a cell signaling molecule. This study suggests that HDL particle-reconstituted GPCRs can provide an effective platform method for the identification of GPCR binding partners coupled with a mass spectrometry-based proteomic analysis.

## Introduction

GPCRs are the largest family of membrane proteins in the human genome and perform vital signaling functions in vision, olfactory perception, and signal transduction processes in the metabolic, endocrine, neuromuscular and central nervous systems [1]. All GPCRs share a common seven-transmembrane (TM) α-helical structure with an extracellular N-terminus and an intracellular C-terminus. Agonists bind on the extracellular side of the receptors, which promotes conformational changes in the TM segments and associated intracellular regions. These conformational changes lead to the interaction and activation of heterotrimeric G proteins (α, β and γ subunits) [1]. However, heterotrimeric G proteins are not the only proteins that bind to GPCRs, and growing evidence indicates a variety of other proteins may physically and functionally associate with GPCRs [2], [3]. A large set of recent studies demonstrate that many other intracellular molecules interact with GPCRs to regulate G-protein-independent signaling, desensitization, internalization, and resensitization [2], [3], [4], [5].

The majority of neuro-hormonal signals to the heart are mediated by GPCRs [6], [7], [8]. Occasionally these signals become pathogenic; for example chronically elevated sympathetic activity stimulating β-adrenergic receptors (βARs) is associated with heart failure progression and mortality [9], [10]. Previous studies suggest that chronic stimulation of the β_1_AR plays a major role in the pathogenesis of dilated cardiomyopathy, while chronic stimulation of β_2_ARs is protective [10]. Due to the adverse effects of chronic activation of β_1_ARs, β-blockers have been widely used for heart failure management. Although βARs have been among the most extensively studied member of GPCRs in the heart, little is known about the signaling pathways that mediate the pathologic response to chronic β_1_AR stimulation or the protective effects of β_2_AR stimulation. Therefore, identification of βAR-mediated signaling pathways will provide better understanding for the development of therapeutic targets for heart failure.

To date, yeast-2-hybrid overlay technologies or pull-down assay followed by mass spectrometry-based protein identification have been used to identify protein-protein interaction [11], [12]. Mass spectrometry has become the method of choice for the identification, quantification, and detailed primary structural analysis of protein components in complex mixtures [11], [13], [14], [15]. However, the application of mass spectrometry in the identification of GPCR-interacting proteins has been limited due to the challenges of working with membrane proteins and the low abundance of GPCRs in native tissue [16], [17].

To address these challenges, we developed a new approach for identifying interacting proteins by preparing GPCRs within high-density lipoprotein (HDL) particles, where GPCRs are in a more membrane-like environment when compared to a detergent micelle. An HDL particle is composed of a dimer of apolipoprotein A-I (ApoA-I) surrounding a planar bilayer of about 160 phospholipids in which GPCRs are easily reconstituted *in vitro* [reconstituted HDL (rHDL)] [18]. Electron microscopy images of these particles showed the uniform disk-shaped structure (10–12 nm in diameter and thickness of 40 Å, the same thickness of a plasma membrane) [18]. Previous studies demonstrate that HDL particle-reconstituted β_2_AR (β_2_AR•rHDL) is monomeric and fully functional by virtue of its capacity to support both high-affinity agonist binding and rapid agonist-mediated nucleotide exchange of G proteins [18].

In this study, we used the β_2_AR•rHDL as bait for the identification of β_2_AR-interacting proteins in heart cytosol to gain insights into the β_2_AR-mediated signaling pathways in the heart. β_2_AR-interacting proteins present in the adult rat heart cytosol were identified using β_2_AR•rHDLs and bioinformatic analysis. The identified molecules suggest some novel β_2_AR signaling pathways in the heart, which could provide insight into the non-canonical roles played by the β_2_AR in the heart.

## Materials and Methods

### Ethics Statement

The use of animals for the experiments followed Stanford University guidelines and all experiments involving animals were approved by the Stanford University Administrative Panel on Laboratory Animal Care.

### Materials

All materials were purchased from Sigma Aldrich (St. Louis, MO) unless otherwise indicated. *Sf*9 insect cells, insect cell culture media and transfection reagents were obtained from expression systems (Woodland, CA). Dodecylmaltoside was from Affymetrix (Santa Clara, CA). Palmitoyl-oleoyl-glycero-phosphocholine and palmitoyl-oleoyl-phosphatidylglycerol were from Avanti Polar Lipids (Alabaster, AL). Complete protease inhibitor cocktail was from Roche (Indianapolis, IN). Ni-NTA resin was made by using Chelating sepharose fast flow (GE Healthcare Biosciences, Pittsburgh, PA) according to the manufacture’s instruction.

### Heart Cytosol Preparation

Adult Sprague Dawley rat hearts were homogenized in buffer A (25 mM HEPES, 140 mM KCl, 12 mM NaCl, 0.8 mM MgSO_4_, 1 mM EDTA, pH 7.4) containing complete protease inhibitor cocktail. Crude homogenate was centrifuged for 10 min at 1000 g, and the supernatant was centrifuged again for 30 min at 18,000 g at 4°C. The supernatant was collected as cytosol, and the protein concentration was adjusted to 10 mg/ml.

### β_2_AR and ApoAI Preparation

β_2_AR was prepared as previously described. Briefly, N-terminally Flag-tagged β_2_AR was expressed in *Sf9* insect cells using recombinant baculovirus [19]. *Sf9* cell membranes were solubilized in dodecylmaltoside, and the β_2_AR was purified by sequential Flag-specific M1 antibody and ligand affinity chromatography. Wild-type His-tagged human ApoA-I was expressed and purified from *E. coli* as previously described [18]. The purity of purified β_2_AR and ApoAI was tested by SDS-PAGE and coomassie staining (Figure S1). Figure S1 shows that the purified samples do not have proteins other than β_2_AR or ApoAI.

### HDL Particle Formation

β_2_AR was reconstituted into rHDL as previously described [18]. Briefly, a mixture of palmitoyl-oleoyl-glycero-phosphocholine and palmitoyl-oleoyl-phosphatidylglycerol were used in combination (3∶2 molar ratio) to mimic the zwitterionic environment of a cell membrane. Lipids were solubilized with HNE buffer (20 mM HEPES, 100 mM NaCl, 1 mM EDTA, pH7.5) plus 50 mM Cholate. An rHDL reconstitution consisted of the following with final volume of 1.3 ml: 24 mM cholate, 8 mM lipid, and 100 µM apoA-I in HNE buffer. For receptor reconstitution in rHDL particle, 2 µM of β_2_AR was added. After incubation for 2 hrs on ice, samples were subjected to BioBeads (BioRad, Hercules, CA) to remove detergents, resulting in the formation of rHDL. β_2_AR•rHDL were subsequently purified from receptor-free empty rHDL and immobilized by 100 µl M1-anti-FLAG immunoaffinity resin. Empty rHDL for the negative control was prepared by same procedure but without β_2_AR.

### Pull-down β_2_AR-interacting Proteins from Heart Cytosol

β_2_AR•rHDL (106 µg of β_2_AR) was immobilized to Flag-specific M1 resin (100 µl) by mixing β_2_AR•rHDL and M1 resins for 2 hrs at room temperature as described above. Three ml of prepared heart cytosol (10 mg/ml) with 4 mM CaCl_2_ was added and incubated overnight at 4°C. The supernatant were removed and resins were washed 7 times with ice cold 1 ml buffer B (25 mM HEPES, 140 mM KCl, 12 mM NaCl, 0.8 mM MgSO_4_, 2 mM CaCl_2_, pH7.4). β_2_AR•rHDL and β_2_AR•rHDL-interacting proteins were eluted by 200 µl elution buffer (20 mM HEPES, 100 mM NaCl, 0.2 mg/ml FLAG peptides and 8 mM EDTA, pH 7.5). With this elution condition, β_2_AR•rHDL and β_2_AR•rHDL-interacting proteins are effectively eluted, but M1 antibody remains on the beads. The eluted samples were concentrated using speedvac by reducing the volume down to 50 µl.

### Negative Control Experiments

**Figure 1:**
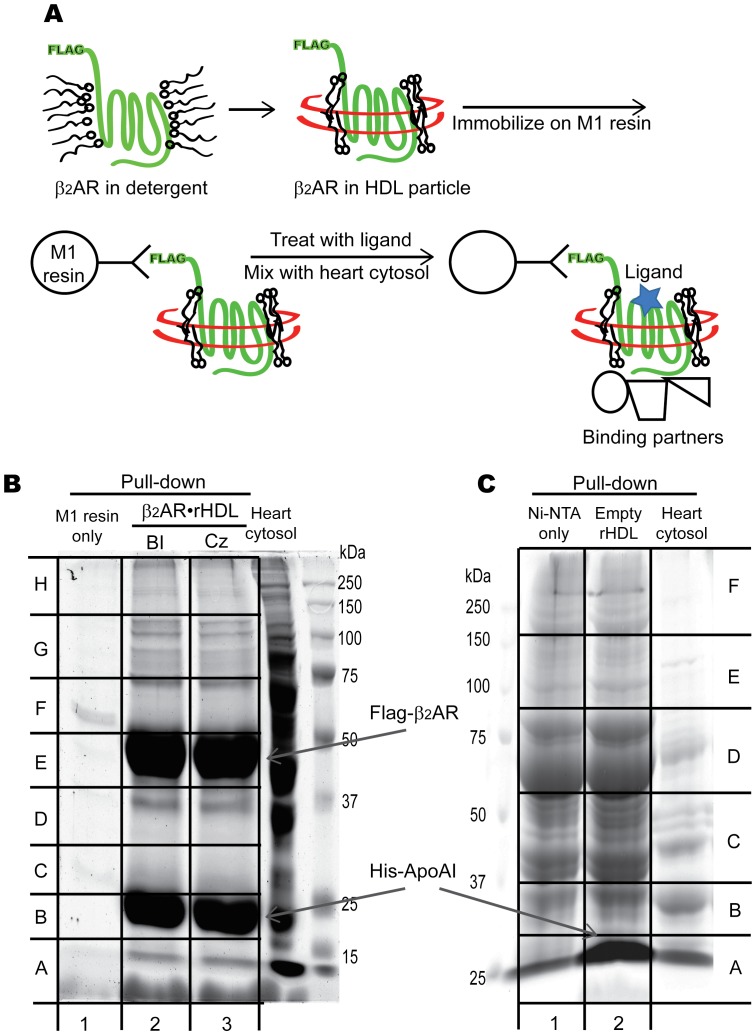
Co-immunoprecipitation of β_2_AR-interacting proteins in the adult rat heart cytosol. A) Reconstitution process of β_2_AR into HDL particles. B) Coomassie-stained SDS-PAGE gel of β_2_AR•rHDL-interacting proteins. C) Coomassie-stained SDS-PAGE gel of empty HDL-interacting proteins. BI: 50 µM BI-167107, Cz: 50 µM Carazolol. Gel pieces (1A through 3H for Figure 1B, and 1A through 2F for Figure 1C) were cut out for mass spectrometry-based protein identification.

We used two different negative controls; M1 resin control and empty rHDL control. M1 resin control was used to identify proteins that bind nonspecifically to M1 resin. For the M1 resin control, empty M1 resin (100 µl) was incubated with heart cytosol (3 ml of 10 mg/ml) overnight at 4°C followed by the procedure as described above. M1 resin control and β_2_AR•rHDL pull-down samples were run on the SDS-PAGE gel and stained with GelCode Blue Stain Reagent (Pierce, Rockford, IL) (Figure 1B). The first lane of Figure 1B indicates M1 resin control.

Empty rHDL control was used to identify proteins that bind to either ApoAI or to lipids in the rHDL. Empty rHDL was prepared as described above without adding β_2_AR and immobilized on 100 µl Ni-NTA resin by using the His-tag on ApoAI. Heart cytosol (3 ml of 10 mg/ml) was incubated with empty rHDL-immobilized Ni-NTA resin or Ni-NTA resin overnight at 4°C. Ni-NTA resin was used to discriminate the proteins that nonspecifically bind to the Ni-NTA resin. The resins are washed extensively with buffer B, and bound proteins were eluted with buffer B containing 200 mM imidazole. Empty rHDL-immobilized Ni-NTA resin control and Ni-NTA resin samples were run on the SDS-PAGE gel and stained with GelCode Blue Stain Reagent (Figure 1C). The first lane of Figure 1C indicates empty Ni-NTA resin samples, and the second lane indicates Empty rHDL-immobilized Ni-NTA resin control.

### MS and Identification

Forty five µl of eluted samples were loaded onto 10% polyacrylamide gel and stained with GelCode Blue Stain Reagent. Gel lanes (Figure 1B and 1C) were cut and submitted to the Vincent Coates Foundation Mass Spectrometry Laboratory at Stanford University for in-gel tryptic digestion and protein identification by mass spectrometry. Scaffold 3 (Proteome Software Inc., Portland, OR) was used to validate MS/MS-based peptide and protein identifications. Peptides were identified from MS/MS spectra by searching for the IPI *Rattus norvegicus* database using the Mascot search algorithm (www. matrixscience.com). The following parameters were used: trypsin specificity, cysteine carbamidomethylation as a fixed modification. Protein identifications were accepted if they could be established at >95.0% probability and contained at least two unique identified peptides. Protein probabilities were assigned by the Protein Prophet algorithm. Using these stringent identification parameters, peptide false detect rate was 0.2%, and protein false detect rate was 0.1%.

### Data Anlaysis

Bioinformatics analysis of molecule function classification and canonical pathway analysis was performed using of Ingenuity Pathways Analysis (Ingeunity® Systems, www.ingenuity.com). The Functional Analysis identified the biological functions that were most significant to the data set. Right-tailed Fisher’s exact test was used to calculate a p-value determining the probability that each biological function assigned to that data set is due to chance alone. Canonical pathways analysis identified the pathways from the Ingenuity Pathways Analysis library of canonical pathways that were most significant to the data set. The significance of the association between the data set and the canonical pathway was measured in 2 ways: 1) A ratio of the number of molecules from the data set that map to the pathway divided by the total number of molecules that map to the canonical pathway is displayed. 2) Fisher’s exact test was used to calculate a p-value determining the probability that the association between the genes in the dataset and the canonical pathway is explained by chance alone.

### Western Blot

Five µl of eluted samples or 1 µl of cell lysates were separated by 10% SDS–PAGE, and transferred to a PVDF membrane. Blots were blocked with 5% nonfat dry milk for 1hr at room temperature, and then incubated with a primary antibody for 2 hrs at room temperature, followed by incubation with a IR dye-labeled secondary antibody (Rockland Immunochemicals, Gilbertsville, PA) for 1hr at room temperature. The signal was visualized with Odyssey imaging systems (LI-COR biosciences, Lincoln, NE).

## Results

### Pull-down of β_2_AR-interacting Proteins

To isolate proteins that interact with the β_2_AR in the heart, we reconstituted purified β_2_AR in rHDL and immobilized it on Flag-specific M1 resin. Immunoprecipitation by M1 antibody is beneficial because proteins can be eluted without disrupting the interaction between M1 antibody and the resin, so there is no M1 IgG protein in the eluted sample. Please note that there is no IgG band at 50 kDa or 25 kDa in the first lane of Figure 1B. The bands at 50 kDa and 25 kDa in the second and third lanes of Figure 1B are β2AR and ApoAI respectively. β_2_AR•rHDL immobilized on M1 resin was occupied by either 50 µM of the full agonist BI-167107 (BI) or 50 µM of the inverse agonist Carazolol (Cz), and then incubated with adult rat heart cytosol (Figure 1A). β_2_AR•rHDL and interacting proteins were eluted, separated on SDS-PAGE, and stained with GelCode Blue Stain Reagent (Figure 1B). To exclude proteins non-specifically bound to the M1 resin, heart cytosol was incubated with M1 resin alone (lane 1, Figure 1B) (See “Materials and Methods” for details). To exclude proteins bound to rHDL, empty rHDL-immobilized Ni-NTA resin was incubated with heart cytosol (lane 2, Figure 1C). The proteins that were nonspecifically bound to Ni-NTA resin were eliminated by including Ni-NTA resin control (lane 1, Figure 1C). Therefore, BI-occupied β_2_AR-interacting proteins were defined as proteins identified in [((lane 2 of Figure 1B) – (lane 1 of Figure 1B)) – (lane2 of Figure 1C) – (lane 1 of Figure 1C))]. Similarly, Cz-occupied β_2_AR-interacting proteins were defined as proteins identified in [((lane 3 of Figure 1B) – (lane 1 of Figure 1B)) – (lane2 of Figure 1C) – (lane 1 of Figure 1C))]. Gel pieces were cut out with no gaps from each lane (1A through 3H of Figure 1B and 1A through 2F of Figure 1C) and subjected to in-gel trypsin digestion. The tryptic digests were analyzed through Thermo LTQ-Orbitrap Velos ETD LC-MS.

**Figure 2:**
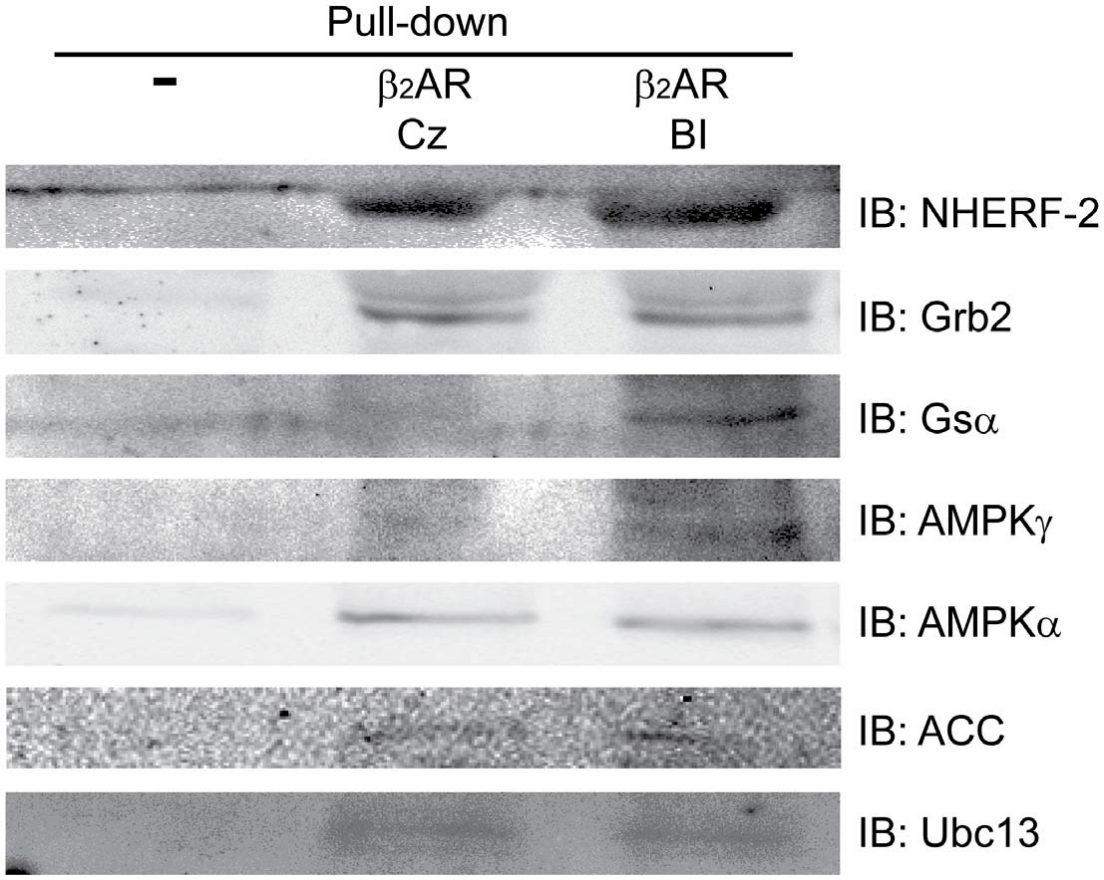
Validation of identified proteins by Western blotting. β_2_AR•rHDL-pull down samples were run on SDS-PAGE gel, transferred to PVDF membrane, and analyzed by Western blotting using antibodies specific for each binding partners. The figures are the representative of at least two independent experiments. BI: 50 µM BI-167107, Cz: 50 µM Carazolol.

### Identification of β_2_AR-interacting Proteins by MS

A total of 521 proteins were identified from the gel pieces shown in Figure 1B, and 265 proteins were identified from the gel pieces shown in Figure 1C (Table S1). After subtracting proteins that were found in the control experiments as described above, 327 proteins were identified specifically in β_2_AR•rHDL pull-down samples (Table S2). The majority of proteins (210 proteins) were found in both the BI-occupied and the Cz-occupied samples. Eighty-three proteins were detected only the in the BI-occupied sample, and 32 proteins were specific for the Cz-occupied sample (Table S2). Protein false detect rate was 0.1% (See Materials and Methods). The majority of proteins were detected at the expected molecular weight range (Table S2, and Table 1 for selected proteins). Five of the identified proteins are known to interact with β_2_AR based on protein-protein interaction databases (BioGrid, MINT, IntAct, HPRD and MIPS) (Table S2, Table S3 and Table 1). The interaction of subset of newly identified-proteins with the β_2_AR was further confirmed by Western Blotting (See below).

**Table 1:** Summary of selected identified proteins.

			% Sequence coverage		Gel slice #	
Protein Name	Accession #	MW	BI	CZ	BI	Cz
**Known β_2_AR-interacting proteins**						
Gsα	IPI00199872	46 kDa	25.4	0.0	D/E	-
Grb2	IPI00203630	25 kDa	0.0	12.0	-	B
NHERF-1	IPI00200898	39 kDa	28.1	25.6	D/E/F	E/F
NHERF-2	IPI00558908	35 kDa	21.8	25.0	A/C/D/E/G/H	A/C/D/E
Serine/threonine-protein phosphatase 2B, catalytic subunit alpha	IPI00201410	59 kDa	0.0	5.8	-	F
**Novel proteins**						
AMPKα2	IPI00201424	62 kDa	29.7	31.5	F	F
AMPKγ1	IPI00196645	37 kDa	60.0	57.3	D	D
AcetylCoA carboxylase 2 (ACC)	IPI00190024	276 kDa	10.6	9.1	H	H
Ubiquitin-conjuating enzyme E2 D3 (UBC13)	IPI00192159	17 kDa	20.1	0.0	A	-

### Validation of the Identified Proteins by Western Blotting

To validate the MS analysis results, we performed Western Blot analysis on select proteins (Table 1 and Figure 2). Both known β_2_AR-interacting proteins (NHERF-2, Grb2 and Gsα) and novel β_2_AR-interacting proteins (AMPKγ, AMPKα, ACC and Ubc13) were selected for validation by Western Blotting (Figure 2). Interestingly, both in the MS analysis (Table S2 and Table 1) and on the Western Blot (Figure 2), Gsα was only identified in the agonist-occupied pull-down sample. In contrast, Grb2 and Ubc13 were found both in agonist and inverse agonist occupied pull-downs by Western Blotting (Figure 2) but only in inverse agonist-occupied samples in the proteomic analysis (Table S2 and Table 1). These proteins were not detected in M1 resin control (lane 1, Figure 2), empty rHDL-immobilized Ni-NTA or empty Ni-NTA negative controls (Data not shown).

### Bioinformatics Analysis

**Figure 3:**
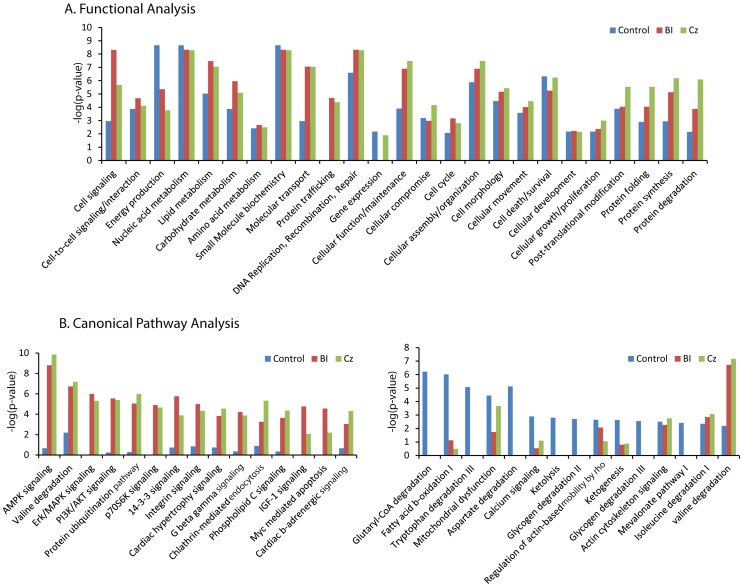
Bioinformatic analyses of identified proteins. A) Functional distribution of β_2_AR-interacting proteins in the adult rat heart cytosol. Total list of functional distribution of identified proteins are summarized in Table 2. B) Canonical signaling pathway analysis of β_2_AR-interacting proteins (left) compared to control (right). Only the top 15 canonical signaling pathways are presented. BI: 50 µM BI-167107, Cz: 50 µM Carazolol.

Proteins from β_2_AR•rHDL pull-downs and control pull-downs were assigned broad classification based upon known or predicted functions by using Ingenuity Pathway Analysis software. Proteins with unknown functions were omitted from this classification, and proteins with multiple functions were assigned to both groups. Proteins were grouped into 25 functional groups; Cell signaling, Cell-to-cell signaling/interaction, Energy production, Nucleic acid metabolism, Lipid metabolism, Carbohydrate metabolism, Amino acid metabolism, Small molecule biochemistry, Molecular transport, Protein trafficking, DNA replication/recombination/repair, Gene expression, Cellular function/maintenance, Cellular compromise, Cell cycle, Cellular assembly/organization, Cell morphology, Cellular movement, Cell death/survival, Cellular development, Cellular growth/proliferation, Post-translational modification, Protein folding, Protein synthesis, Protein degradation (Figure 3A and Table 2). Interestingly, molecules involved in cell signaling, molecular transport, protein trafficking, and protein degradation are predominant in the β_2_AR•rHDL pull-downs compared to the control sample.

**Table 2:** Cellular Functions of Identified Proteins.

Functions	Identified Proteins
Cell Signaling	ALOX15, NRAS, RAB3A, CFL1, RRAD, ATP2A1, RAB7A, ATP2A3, LOC643751, NOS3, RAP1A, KPNB1, TGM2, GNB1, PPP2CB, HP, RRAS2, CADPS, GNAO1, EEF1A1, DNAJA3, YWHAQ (includes EG:22630), IPO5, NME2, RHEB, (*STAT4, GNAI2, GNAS, RHOG, PRKAA1, LOC643751, YWHAE, STAT1)*, (**HK1, MTOR, ERP44, GRB2, RSU1, PPP3CA, SOD1, SOD2**)
Cell-To-Cell Signaling and Interaction	EHD1, ALOX15, CFL1, RAB21, APOA1, RRAD, CD36, INPPL1, NOS3, RAP1A, GNB1, ROCK2, TGM2, PLA2G6, CAPN1, TXN, MARCKS (includes EG:4082), CLIC4, AKR1B1, HSPB1, FGA, (*GNAI2, STAT4, GNAS, PSME1, SERPINC1, C3, MAP2K3, STAT3, LOC643751, STAT1, Ube2n, YWHAG, TNS1*), (**SLK, MTOR, GRB2, RSU1, SOD1, SOD2**)
Energy Production	DLD, CD36, PRKAA2, NOS3, LONP1, MSRA PRDX5 UBA1, (*ECHS1, ECI1*)
Nucleic AcidMetabolism	NRAS, ACACB, CD36, RAB7A, NOS3, TGM2, KPNB1, GNB1, RRAS2, GNAO1, EEF1A1, DLD, PRKAA2, MLYCD, HMGCL, ALDH2, NME2, AKR1B1, ALDH6A1, PRDX5, UBA1, LONP1, IPO5, (*GNAI2, GNAS, AMPD3, GPD1, STAT3*), (**ADSL, GFPT1, SOD1**)
Lipid Metabolism	QKI, APOA1, PTPMT1, CRAT, SH3GLB1, LIPE, INPPL1, NOS3, ROCK2, GNB3, PTGES2, HBB (includes EG:3043), GPLD1, PRKAA2, MGLL, MARCKS (includes EG:4082), GPX4, HMGCL, PITPNB, PLA2G16, ALOX15, ACACB, RAB5A, RRAD, RAB7A, CD36, PCYT1A, PLA2G6, HP, ARF1, GNAO1, CPT2, DLD, EEF1A1, AKR1B1, MAP4, TPP1, MLYCD, (*GNAI2, GNAS, SERPINC1, SDHB, LPIN1, C3, FABP4, PCCA, YWHAH, ECHS1, ECI1, SUCLG1, PRKAA1*), (**SDHA, MTOR, ACBD3, SOD1, SOD2**)
Carbohydrate Metabolism	IDH3G, APOA1, PTPMT1, PPP1CB, LIPE, CRAT, SH3GLB1, FN3K, INPPL1, NOS3, PRKAG1, GNB1, ROCK2, HK2, GPLD1, PRKAA2, MGLL, PPM1A, IDH3A, GPX4, MARCKS (includes EG:4082), PLA2G16, ACACB, AKR1B1, ALDH2, SLC9A3R1, RAB5A, RRAD, COQ3, CD36, PFKM, PCYT1A, PLA2G6, ARF1, EEF1A1, MLYCD, (*C3, GPD1, PRKAA1, STAT3, PGAM2, ACO1, FABP4*), (**HK1, MTOR, GFPT1, GRB2, SOD1, SOD2**)
Amino AcidMetabolism	PPP2CB, PPP2R1A, PPP2CA, PPP1CB, PPM1A, NOS3, ALDH6A1, MSRA, PCYT1A, (*MTHFD1,PCCA*), (**SLK, HK1, MTOR, PPP2R2A, F2, PPP3CA, SOD1**)
Small Molecule Biochemistry	QKI, PPP2CA, SH3GLB1, LIPE, NOS3, GNB1, ROCK2, GNB3, PTGES2, HBB (includes EG:3043), CADPS, MGLL, PPM1A, GPX4, PITPNB, ACACB, RRAD, PFKM, PPP2CB, PLA2G6, PPP2R1A, GNAO1, DLD, CPT2, MLYCD, APOA1, IDH3G, PTPMT1, PPP1CB, CRAT, FN3K, INPPL1, PRKAG1, TGM2, HK2, GPLD1, PRKAA2, IDH3A, MARCKS (includes EG:4082), HMGCL, LONP1, IPO5, PLA2G16, ALOX15, NRAS, RAB5A, COQ3, CD36, RAB7A, PCYT1A, KPNB1, ARF1, HP, ALDH2, ALDH6A1, AKR1B1, PRDX5, MSRA, NME2, MPST, MAP4, UBA1, RRAS2, EEF1A1, TPP1, (*AMPD3, SDHB, GPD1, C3, YWHAZ, STAT3, MTHFD1, GNAI2, STAT4, GNAS, GPX3, SERPINC1, LPIN1, PRKAA1, AKR7A2, PCCA, APRT, SUCLG1, FABP4, YWHAH, ACO1, ECHS1, ECI1, PGAM2, STAT1*), (**SLK, SDHA, HK1, ADSL, MTOR, GFPT1, ERP44, PPP2R2A, GRB2, ACBD3, PPP3CA, SOD1, SOD2**)
Molecular Transport	RAB1A, APOA1, RAB2A, ATP2A1, CRAT, LIPE, PPP1CB, INPPL1, NOS3, ROCK2, HK2, HBB (includes EG:3043), COQ7, CADPS, PRKAA2, PPM1A, MGLL, PITPNB, ALOX15, NRAS, ACACB, RAB3A, RAB5A, CFL1, TIMM44, RRAD, RAB10, RAB7A, CD36, ATP2A3, PCYT1A, KPNB1, PLA2G6, HP, ARF1, ARF3, GNAO1, CPT2, EEF1A1, DLD, RAB3GAP2, MLYCD, PRDX5, AKR1B1 ALDH2, NME2, CLIC4, RAB6A, (*GNAI2, GNAS, SERPINC1, LPIN1, C3, YWHAZ, ACO1, YWHAE, FABP4, APRT, NDRG2, YWHAH, PGAM2, ECI1, STAT3*), (**GFPT1, MTOR, ERP44, GRB2, ACBD3, PPP3CA, SOD1, SOD2**)
Protein Trafficking	RAB1A, RAB5A, TIMM44, CFL1, RAB2A, CD36, RAB10, RAB7A, KPNB1, ARF1, ARF3, RAB3GAP2, NME2, RAB6A, (*YWHAE, YWHAG*), (**MTOR, SOD1, SOD2**)
DNA Replication/Recombination/Repair	NRAS, CFL1, RAB7A, TGM2, KPNB1, GNB1, DYNLL1, RRAS2, GNAO1, PRKAA2, TXN, HSPB1, NME2, SELENBP1, UBA1, LONP1, IPO5, (*GNAI2, GNAS, AMPD3, GPD1, PIN1, STAT1*), (**SOD1, SOD2**)
Gene Expression	PTGES2, PPP2CA, (*STAT4, GNAS, SERPINC1, YWHAZ, MAP2K3, STAT3, PIN1, STAT1, ELAVL1, YWHAE, ACO1, TCEB1*), (**GFPT1, MTOR, PSMC3**)
Cellular Function and Maintenance	EHD1, NRAS, RAB5A, RAB3A, ATP2A1, CD36, RAB7A, LIPE, NOS3, TGM2, ROCK2, DYNC1H1, PLA2G6, FIS1, HP, DYNLL1, EHD4, CADPS, EHD2, PRKAA2, TXN, DNM1L, (*GNAI2, STAT4, GNAS, SERPINC1, LPIN1, C3, SEC23IP, PRKAA1, STAT3, STAT1, LOC643751*), (**HK1, MTOR, PLS3, GRB2, RSU1, PPP3CA**)
Cellular Compromise	EHD1, ALOX15, RAB1A, CFL1, RRAD, ATP2A1, NOS3, ROCK2, FIS1, DYNLL1, HP, DSTN, DLD, ALDH2, CLIC4, AKR1B1, TXN, DNM1L, (*C3, OPA1, SORBS2, STAT3*), (**SLK, SDHA, MTOR, COX4I1**)
Cell Cycle	ARL8B, CUL5, PPP2CA, CUL4A, CUL1, DDX3X, LIPE, LOC643751, ROCK2, DSTN, PPM1A, TXN, MARCKS (includes EG:4082), PLA2G16, ALOX15, ACACB, NRAS, CFL1, RRAD, DDB1, FIS1, PPP2CB, SIRT2, AKR1B1, MAP4, CAPN2, DNM1L, (*GNAI2, STAT4, GNAS, C3, OPA1, MAP2K3, PIN1, STAT3, STAT1, LOC643751, YWHAE, NPEPPS, ELAVL1*), (**MTOR, PPP2R2A, GRB2, PPP3CA, SOD1, SOD2**)
Cellular Assembly and Organization	RAB1A, RAB12, RAB2A, APOA1, ATP2A1, SH3GLB1, LIPE, INPPL1, RAB5B, LOC643751, NOS3, ROCK2, GNB1, DYNLL1, HK2, CADPS, RAB5C, EHD2, DSTN, ARL1, DNAJA3, SLC9A3R2, TXN, MARCKS (includes EG:4082), EHD1, RAB3A, CFL1, MAP4, HSPB1, SELENBP1, CLIC4, RAB5A, RAB7A, PCYT1A, FIS1, ARF1, CAPNS1, ARF3, CAPN1, SAR1A, GNAO1, EEF1A1, TPP1, DNM1L, RAB6A, (*LRBA, C3, SEC23IP, YWHAZ, BASP1, STAT3, LOC643751, IQGAP1, GNAI2, GNAS, SERPINC1, NOL3, LPIN1, RHOG, OPA1, SORBS2, PIN1, NFS1, YWHAG, TNS1*), (**HK1, MTOR, PLS3, GRB2, PPP3CA, SOD1, SOD2**)
Cell Morphology	EHD1, NRAS, RAB1A, CFL1, RRAD, ATP2A1, SH3GLB1, LIPE, NOS3, LOC643751, ROCK2, FIS1, HK2, FLNC, MARCKS (includes EG:4082), GPX4, CLIC4, HSPB1, MAP4, DNM1L, (*SEC23IP, YWHAZ, BASP1, STAT3, LOC643751, IQGAP1, STAT4, GNAS, SERPINC1, LPIN1, NOL3, RHOG, PRKAA1, OPA1, SORBS2, MAP2K3, PIN1, NFS1,YWHAE, YWHAG, CRIP2, TNS1*), (**HK1, MTOR, GRB2, SOD1, SOD2**)
Cellular Movement	CUL5, APOA1, MYLK3, LIPE, DDX3X, NOS3, LOC643751, HNRNPK, ROCK2, TGM2, DNAJA3, FGB, MGLL, SLC9A3R2, TXN, MARCKS (includes EG:4082), EHD1, NRAS, RAB21, SLC9A3R1, CFL1, CD36, RAP1A, PLA2G6, HP, CAPNS1, RRAS2, CAPN1, GNAO1, CAPN2, AKR1B1, CLIC4, HSPB1, NDRG2, NME2,FGA, (*GNAI2, GNAS, SERPINC1, C3, RHOG, MAP2K3, PIN1, STAT3, IQGAP1, LOC643751, STAT1, TNS1*)
Cell Death/Survival	CUL5, QKI, APOA1, PPP2CA, CUL4A, CUL1, ATP2A1, BAG3, SH3GLB1, LIPE, DDX3X, INPPL1, NOS3, LOC643751, ROCK2, CIAPIN1, GNB1, TGM2, DYNLL1, HK2, PRKAA2, DNAJA3, SLC9A3R2, PPM1A, TXN, GPX4, RHEB, PLA2G16, ALOX15, NRAS, CD36, SIRT3, PFKM, PPP2CB, PLA2G6, FIS1, PPP2R1A, TUBA1A, CAPNS1, RRAS2, SIRT2, CAPN1, GNAO1, EEF1A1, TPP1, CLIC4, MAP4, UBA1 HSPB1, AKR1B1, ALDH2, MAP4, NME2, PRDX5, SELENBP1, UBA1, MSRAD, NM1L, (*SDHB, C3, YWHAZ, STAT3, LOC643751, ELAVL1, STAT4, GNAS, SERPINC1, NOL3, PRKAA1, OPA1, MAP2K3, SORBS2, PIN1, STAT1*), (**SDHA, SLK, HK1, MTOR, GFPT1, PPP2R2A, GRB2, BAT3, PPP3CA, SOD1, SOD2**)
Cellular Development	EHD1, ALOX15, NRAS, RRAD, LIPE, NOS3, LOC643751, ROCK2, FIS1, PLA2G6, PPP2R1A, SIRT2, FLNC, PPM1A, MARCKS (includes EG:4082), ALDH6A1, CLIC4, NME2, SELENBP1, NDRG2, UBA1, AKR1B1, FGA, (*STAT4, GNAS, LPIN1, C3, BASP1, MAP2K3, PIN1, STAT3, STAT1, LOC643751, FABP4, Ube2n, FABP4, YWHAG, ELAVL1*), (**MTOR, GRB2, SOD1, SOD2**)
Cellular Growth and Proliferation	RAB1A, PRKAB1, PPP2CA, APOA1, CUL1, DDX3X, NOS3, LOC643751, HNRNPK, ROCK2, ARL1, DNAJA3, PPM1A, SLC9A3R2, TXN, GPX4, ALOX15, NRAS, SLC9A3R1, RRAD, PSME2, DDB1, DYNC1H1, PLA2G6, ARF1, PPP2R1A, PSMC1, CAPNS1, SIRT2, CAPN1, PSMD2, NME2, NDRG2, AKR1B1, UBA1, GNAO1, (*STAT4, SERPINC1, RHOG, LPIN1, C3, PRKAA1, MAP2K3, STAT3, PIN1, STAT1, LOC643751, ELAVL1*), (**HK1, MTOR, GRB2, PSMC3, PPP3CA, SOD1, SOD2**)
Post-Translational Modification	CUL5, APOA1, PPP2CA, CUL1, CD36, PPP1CB, SH3GLB1, SIRT3, TTC1, NOS3, DDB1, PPP2CB, PPP2R1A, UGGT1, SIRT2, CAPN1, DNAJA3, CAPN2, PPM1A, ALDH2, MSRA, TXN, (*CAND1, FAM129A, UBE2D2, YWHAZ, STAT1, TCEB1*), (**SLK, ADSL, HK1, GFPT1, MTOR, ERP44, PPP2R2A, PPP3CA, SOD1, SOD2**)
Protein Folding	UGGT1, DNAJA3, SH3GLB1, TXN, TTC1
Protein Synthesis	ALOX15, PRKAB1, PRKAG1, PFKM, TGM2, HBB (includes EG:3043), CAPNS1, CAPN1, PRKAA2, DLD, TPP1, CAPN2, HSPB1, CLIC4, NME2, LONP1, (*KIAA0368, UBE2D2, MAP2K3, UBE2D3 (includes EG:66105*)), (**ADSL, GFPT1, MTOR, ERP44, GRB2, PSMC4, PSMC2, SOD1, SOD2**)
Protein Degradation	ALOX15, CAPNS1, CAPN1, DLD, TPP1, CAPN2, LONP1, (*KIAA0368, UBE2D2, UBE2D3 (includes EG:66105)*), (**MTOR, PSMC4, PSMC2, SOD1, SOD2**)

Normal: proteins identified both in BI-occupied and Cz-occupied β_2_ARr•HDL pull-downs.

(*Italic*): proteins identified in BI-occupied β_2_ARr•HDL pull-downs. (**Bold**): proteins identified in Cz-occupied β_2_AR•rHDL pull-downs.

Identified proteins were also analyzed against canonical pathways by using Ingenuity Pathway Analysis software. The top 15 canonical pathways using the data set of the β_2_AR•rHDL pull-downs or the control sample are presented in Figure 3B. As expected from the known cellular role of β_2_AR, β_2_AR•rHDL pull-downs were biased toward signal transduction pathways (Figure 3B left), whereas controls samples were biased toward metabolic pathways (Figure 3B right).

## Discussion

In the present study, we identified β_2_AR-interacting proteins in rat heart cytosol by using the full-length β_2_ARs reconstituted in the plasma membrane-mimicking HDL particles. To our knowledge, this is the first comprehensive study to investigate GPCR-interacting proteins in the heart or any other primary tissues other than brain. The advantage of reconstituting GPCRs in the HDL particles is that GPCRs are more stable and in a more physiological conformation than detergent-solubilized GPCRs. Furthermore, this approach overcomes the low endogenous expression of the receptor in the heart by using large amount of β_2_AR•rHDL as bait.

Various methods have been used to screen for direct and indirect binding partners of GPCRs. Among those, the affinity isolation/mass spectrometry-based proteomic approach allows the capture and analysis of larger proteome units of protein complexes and can be used for isolating and purifying complexes from cellular and tissue preparations [11], [20]. However, the proteomic analysis of GPCRs has been challenging due to low endogenous expression levels and hydrophobicity of GPCRs. To date, identification of interacting proteins in native tissue has been successful for few GPCRs; including, mGluR5, 5-HT receptors (5-HT-2a, 5HT-2c, and 5-HT4a), and α_2_B-AR [21], [22], [23], [24], [25]. All of these GPCRs were studied in brain tissue where GPCRs and their binding partners are highly expressed, enabling isolation of sufficient quantities of the receptor and associated proteins. Furthermore, studies with 5-HT receptors and α_2_B-AR used c-terminal peptides of the receptors (not the full-length GPCR) as baits [21], [23], [24], [26], [27]. Therefore, those studies cannot identify binding partners that interact with GPCR domains outside of the c-terminus. The present study successfully used full-length β_2_AR as bait and identified binding partners from heart tissue, where the expression level of endogenous β_2_AR is very low.

Although the present study identified β_2_AR-interacting proteins from heart cytosol, there are limitations. First, β_2_AR-interacting membrane proteins cannot be purified because β_2_AR is trapped in the rHDL and detergents cannot be used to solubilize the membrane proteins. GPCRs interact with membrane proteins as well as cytosolic proteins. β_2_AR is also known to interact various membrane proteins (Table S3), but we could not purify these proteins due to the limitations of the system. Second, the results of the present study do not represent proteins that bind to β_2_AR with post-translational modifications (PTMs). GPCRs are known to undergo various PTMs including phosphorylation, ubiquitination, glycosylation and nitrosylation [28], [29], [30]. However, β_2_AR purified from insect cells does not contain the same PTMs vs. β_2_AR from mammalian cells. Therefore, proteins that are known to interact with phosphorylated β_2_AR (e.g. β-arrestins) [31] were not identified in this study. Lastly, as expected, not all previously known β_2_AR interacting proteins were identified in our search (Table S3). This may be due to the intrinsic limitation of mass spectrometry-based protein identification (false-negative detection), low binding affinity of those proteins to β_2_AR, or the artificial environment of β_2_AR•rHDL. Additional studies are required to overcome these limitations; however, we believe that the described method represents an improvement on previously described methods for identifying GPCR-interacting proteins.

Bioinformatic analyses of β_2_AR•rHDL pull-downs showed distinct protein profiles compared to control pull-downs (Figure 3). Functional analysis indicated that a higher percentage of proteins from β_2_AR•rHDL pull-downs are involved in cell signaling and protein trafficking when compared with controls (Figure 3A), suggesting that the identified proteins are not the result of non-specific binding. Canonical pathway analysis used the list of identified proteins to predict relevant signaling pathways and confirmed the difference between β_2_AR•rHDL pull-downs and control pull-down. The majority of pathways from β_2_AR•rHDL pull-downs are signal-transduction related pathways; whereas, most of the top 15 pathways from control pull-downs are related to metabolic proteins that are enriched in the heart (Figure 3B). In addition to the known β_2_AR signaling pathways in the heart (eg. cardiac β-adrenergic signaling, protein ubiquitination, clathrin-mediated endocytosis and G beta gamma signaling), the present study suggests the involvement of the β_2_AR in novel signaling pathways; such as AMPK signaling, PI3K/AKT signaling and integrin signaling pathways (Figure 3). β_2_AR interaction with selected proteins identified in the β_2_AR•rHDL pull-downs were confirmed by co-immunoprecipitation and Western Blot analysis (Figure 2) indicating that the identified proteins are not false-positives. The role of these novel signaling pathways in β_2_AR function in heart physiology and pathology warrants further investigation. Taken together, these bioinformatic analyses confirm the utility of using of GPCR•rHDL as an experimental system to identify GPCR-interacting proteins.

## Supporting Information

Figure S1 **Quality of purified β_2_AR and ApoAI.** Purified β_2_AR (left) and ApoaI (right) were run on SDS-PAGE and visualized with Coomassie staining.
(TIF)

Table S1 **Mass spectrometry-based proteomic identification of proteins in** **Figure 1B and 1C** **.**
(XLSX)

Table S2 **Summary of identified proteins.**
(XLSX)

Table S3 **Known β_2_AR-interacting proteins.**
(DOCX)
