# Supplementary figures and images for: Identification of GPCR-Interacting Cytosolic Proteins Using HDL Particles and Mass Spectrometry-Based Proteomic Approach
Source: PLoS One. 2013 Jan 25;8(1):e54942. doi: 10.1371/journal.pone.0054942 (PMC3556083; doi:10.1371/journal.pone.0054942)

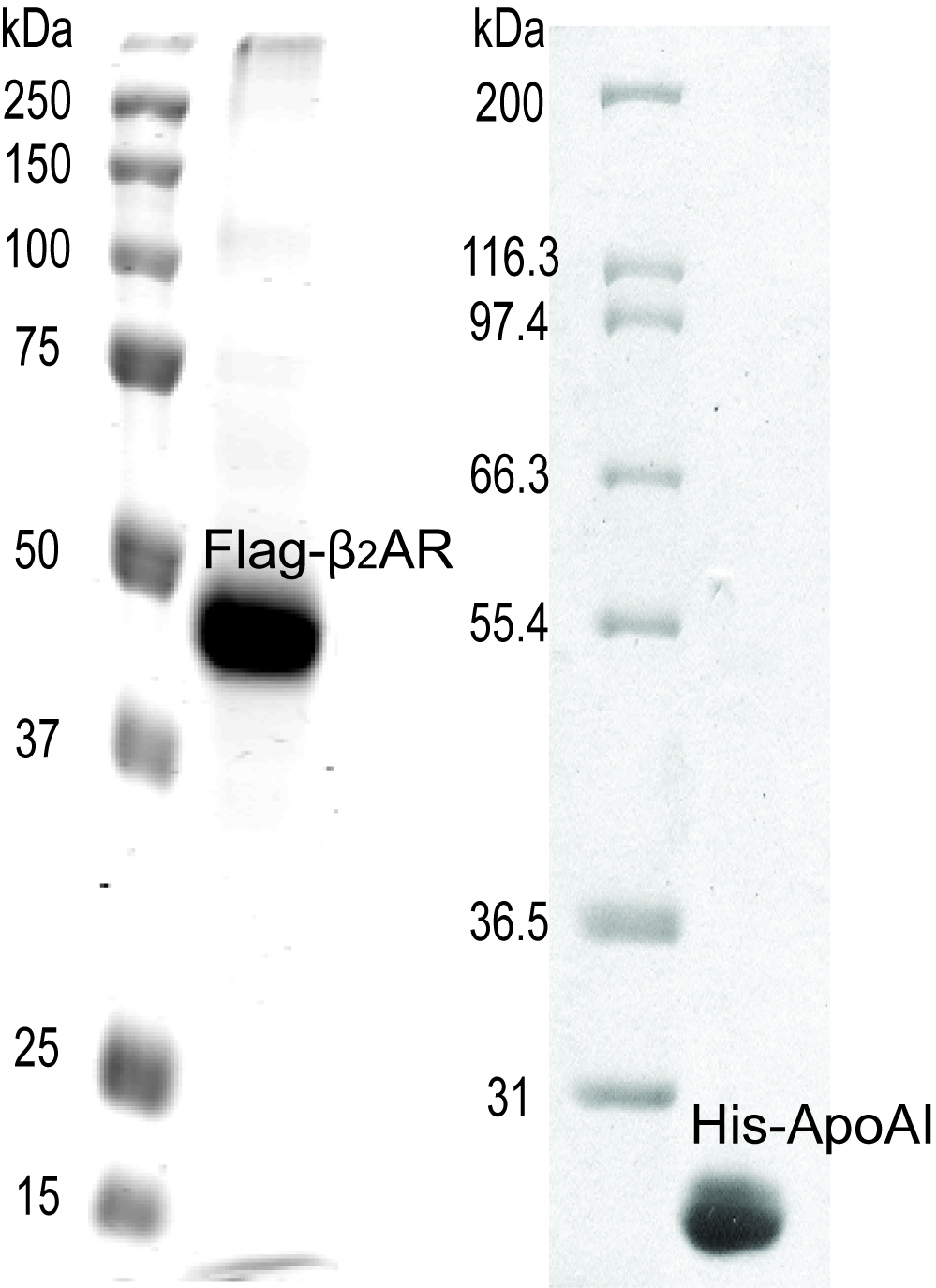

Supplement: Figure S1 — Quality of purified β2AR and ApoAI. Purified β2AR (left) and ApoaI (right) were run on SDS-PAGE and visualized with Coomassie staining. (TIF) [file pone.0054942.s001.tif]
