# Supplementary material for: Identification of GPCR-Interacting Cytosolic Proteins Using HDL Particles and Mass Spectrometry-Based Proteomic Approach
Source: PLoS One. 2013 Jan 25;8(1):e54942. doi: 10.1371/journal.pone.0054942 (PMC3556083; doi:10.1371/journal.pone.0054942)
Supplement: Table S3 — Known β2AR-interacting proteins. (DOCX) [file pone.0054942.s004.docx]

Table S3. Known β_2_AR-interacting proteins

| **Membrane proteins** | **Soluble proteins** |
| --- | --- |
| 5HT4R, ADRB2, ADRB3, GIRK2, GIRK3, GIRK4, GluR1, GluR2, INSR, LAMP2, OPRD | AIF4, AKAP12, AKAP5, ARRB1, ARRB2, ARRC, CNBP, CSK, EI2BA, ERK2, GASP1, GNA11, GNA14, GNA15, GNAS, GNB5, GRB2, GRK2, GRK5, GRK6, IL1B, KPCD, KPCG, MAGI-3, MDM2, NEDD4, NHERF-1, NHERF-2, nNOS, PRKAR2A, PSD-95, SRC, UBC, USP33, ERG, CUL2, CAV3, TCEB1, PPP3CA |

Proteins identified in the present study are red-colored.
